# A predictive model for central nervous system infections in children based on machine learning and clinical diagnostic features

**DOI:** 10.3389/fped.2026.1861527

**Published:** 2026-07-17

**Authors:** Bin Zhou, Feng Liang, Yukun Huang, Shengxin Zhang, Zhiqiang Zhuo, Chunzhi Chen

**Affiliations:** 1 Department of Infectious Diseases, Children’s Hospital of Fudan University (Xiamen Branch), Xiamen Children’s Hospital, Xiamen, Fujian, China; 2 Special Needs Ward, Children’s Hospital of Fudan University (Xiamen Branch), Xiamen Children’s Hospital, Xiamen, Fujian, China; 3 Pediatric Intensive Care Center, Children’s Hospital of Fudan University (Xiamen Branch), Xiamen Children’s Hospital, Xiamen, Fujian, China

**Keywords:** central nervous system infections, children, machine learning, predictive models, shapley additive explanations, support vector machine

## Abstract

**Background:**

Early manifestations of pediatric Central Nervous System Infection (CNSI) lack specificity and are difficult to distinguish from Febrile Seizures (FS). Previous machine learning research has primarily focused on general infection risk stratification, with few studies on discriminative models for pediatric CNSI using initial clinical data.

**Objective:**

To construct an exploratory machine learning-based model for early risk stratification to differentiate pediatric CNSI from FS using single-center retrospective data and to analyze key predictive factors.

**Methods:**

Children hospitalized with fever and convulsions between January 2023 and December 2025 were enrolled. Initial clinical features and laboratory test results were used for univariate screening. After multicollinearity handling (Spearman correlation, |r| ≥ 0.70) and feature importance ranking via Random Forest (RF) and Extreme Gradient Boosting (XGBoost) algorithms, a union set of 10 variables was selected for modeling. The performance of multiple algorithms was compared based on the Area Under the Receiver Operating Characteristic Curve (AUC). Model interpretability was assessed using a nomogram and Shapley Additive exPlanations (SHAP).

**Results:**

A total of 140 children were included (28 in the CNSI group, 112 in the FS group). 10 core features were selected, including: Calcium (Ca) concentration, Lymphocyte Percentage (L%), Serum Albumin (ALB) level, Red Blood Cell (RBC) count, Oxygen Saturation (SO_2_), Lactic Acid (LAC), Neutrophil Percentage (*N*%), Babkinki sign, Headache, and Electroencephalogram (EEG) findings. Among the algorithms, Support Vector Machine (SVM) achieved a relatively high AUC of 0.864 (95% CI: 0.625–1.000) in the internal test set. SHAP analysis indicated that Ca, SO_2_, and L% contributed significantly to the model.

**Conclusion:**

This study developed an exploratory discriminative model for differentiating pediatric CNSI from FS using variables available at initial diagnosis. The model provides preliminary insights for early risk stratification of pediatric CNSI, though its generalizability and clinical utility require further validation through multicenter prospective external studies.

## 1 Introduction

Central nervous system infection (CNSI), an inflammatory disease caused by pathogens such as bacteria, viruses, and fungi invading the brain parenchyma or meninges, remains one of the leading causes of disability and mortality in children under 5 years of age (1–3). In the early stage of the disease, CNSI often presents with“ fever and seizures”, clinical manifestations that highly overlap with those of febrile seizures (FS), posing a substantial challenge to early differential diagnosis (4, 5). Failure to recognize and treat the condition promptly may lead to rapid progression and irreversible neurological damage (6, 7). Therefore, identifying high-risk CNSI patients at an early stage is crucial for improving diagnosis, treatment, and overall prognosis.

Traditional predictive models for childhood CNSI, such as the Children's Bacterial Meningitis Score, the Rochester Criteria, and prognostic nomograms for bacterial meningitis, have demonstrated significant value in the clinical diagnosis of bacterial meningitis (8–10). However, these models largely rely on cerebrospinal fluid (CSF) analysis for confirmation, which is inherently invasive and limits their applicability in primary healthcare settings, thereby hindering the early screening of high-risk CNSI cases. With the advancement of artificial intelligence, developing machine learning-based early prediction models that integrate readily accessible early-stage features holds considerable promise for improving the early identification of CNSI in children.

This study aimed to develop an exploratory model for differentiating pediatric CNSI from FS using machine learning algorithms. By integrating early-stage clinical characteristics and routine laboratory findings available at initial presentation, we constructed a model based on readily accessible variables. This model provides a preliminary reference for early risk stratification of CNSI, with the goal of improving patient prognosis.

## 2 Materials and methods

### 2.1 Study design and subjects

This study employed a single-center, retrospective, exploratory design. The study population comprised pediatric patients hospitalized for “and seizures” at our institution between January 2023 and December 2025.

Inclusion criteria: (1) Aged between 1 month and 18 years; (2) Availability of complete clinical data.

Exclusion criteria: (1) Patients with a history of epilepsy; (2) Children with a history of inherited metabolic disorders; (3) Children with underlying conditions such as brain injury or abnormal brain development; (4) Children who had received treatment at another hospital for the current episode; (5) Children receiving medications such as corticosteroids or immunosuppressants.

Grouping: Patients were assigned to groups based on discharge diagnoses and predefined diagnostic criteria. The case group comprised children with CNSI, including purulent meningitis, viral encephalitis, and meningoencephalitis; The control group comprised children diagnosed with FS during the same period and within the same age range, matched at a ratio of 1∶4.

### 2.2 Collection of clinical data

The following variables were collected:

(1) Demographic data: age, gender, etc.;
(2) Clinical characteristics: fever peak during the course of illness, fever-associated seizures (temperature, type, frequency, duration), Glasgow Coma Scale (GCS), neurological signs (Babkin sign, etc.), accompanying symptoms (headache, vomiting);
(3) Clinical intervention indicators: total length of hospital stay, length of stay in the Pediatric Intensive Care Unit (PICU), whether lumbar puncture was performed, and whether antiviral, intracranial pressure-lowering, sedative, or antibiotic therapy was administered;
(4) Laboratory and Imaging Tests: Complete blood count [neutrophil percentage (*N*%), lymphocyte percentage (L%), red blood cell (RBC) count, etc.], biochemistry [albumin (ALB) levels, calcium (CA) concentration, etc.], Arterial blood gas analysis [Oxygen Partial Pressure (PO_2_), Oxygen Saturation (SpO_2_), Lactic Acid (LAC), etc.], inflammatory markers (C-reactive protein, procalcitonin), electroencephalogram (EEG), and cranial imaging results.

### 2.3 Statistical methods

Data were entered into an Excel spreadsheet using a double-checking method. Statistical analyses were performed using SPSS 25.0, R 4.2.3, and MATLAB 2025b. A two-sided *P*-value < 0.05 was considered statistically significant.

Univariate difference analysis: Continuous variables were first evaluated for normality. Data conforming to a normal distribution are presented as the mean ± standard deviation (x^−^ ± s) and were compared using the independent samples t-test. Non-normally distributed data are expressed as the median (interquartile range) [M (P25, P75)] and were compared using the Mann–Whitney U test. Categorical variables are presented as frequencies (percentages) and were compared using the chi-square (*χ*^2^) test or Fisher's exact test, as appropriate.

Candidate variable selection and collinearity handling: Candidate predictors for the prediction model were restricted to variables readily accessible during the initial clinical evaluation upon admission. Spearman correlation analysis was performed for continuous variables; when |r| ≥ 0.70, the variable with the smaller *P*-value in univariate analysis and stronger clinical interpretability was retained for subsequent modeling. Variable importance was ranked separately using Random Forest (RF) and Extreme Gradient Boosting (XGBoost). Furthermore, the number of variables included in the model was controlled using the Events Per Variable (EPV) principle to avoid an excessive number of variables under small-sample conditions.

Multivariable logistic regression and nomogram construction: A multivariable logistic regression model was fitted using the optimal feature set to estimate the association between variables and the outcome. Odds ratios (OR) and their corresponding 95% confidence intervals (CIs) were calculated. Subsequently, a nomogram was developed based on the regression model to visually quantify the linear combination contribution of each variable for discriminating CNSI from FS.

Machine learning model development: Based on the optimal feature set, binary classification prediction models were constructed and compared across various algorithms, including Logistic Regression (LR), Least Absolute Shrinkage and Selection Operator (LASSO) regression, Ridge regression, Support Vector Machine (SVM), Backpropagation Neural Network (BPNN), RF) and LogitBoost. To mitigate the risk of overfitting, model complexity was controlled for complex algorithms such as RF and LogitBoost by tuning hyperparameters, specifically limiting tree depth, setting minimum leaf node samples, adjusting learning rates, controlling iteration counts, and applying regularization intensity.

Dataset partitioning and model training: The dataset was randomly split into a training set and a testing set at a stratified ratio of 8:2. Hyperparameter optimization was strictly confined to the training set or performed within a cross-validation framework on the training data, ensuring that the testing set remained untouched during model selection. Five-fold stratified cross-validation was implemented within the training set to generate out-of-fold (OOF) predictions, which were used for internal performance assessment and for determining the optimal classification threshold via the Youden index. The testing set was utilized solely for a single, final internal validation assessment.

Model performance evaluation: Model performance was assessed using accuracy, sensitivity, specificity, positive predictive value (PPV), negative predictive value (NPV), F1-score, and the area under the receiver operating characteristic curve (AUC) with its 95% CI. The confusion matrix for the primary models was also reported. The 95% CI for the AUC were calculated using the DeLong method.For the sensitivity analysis regarding class imbalance, the AUC point estimate was derived from the hold-out testing set. However, the lower and upper limits of the 95% CI were updated based on the standard error (SE) obtained from repeated stratified 5-fold cross-validation (repeated 10 times) on balanced training datasets. Specifically, the CI was calculated as “point estimate ± 1.96 × SE” using the DeLong-derived standard error.Furthermore, calibration metrics—including the Brier score and calibration curves—were supplemented where necessary to evaluate the consistency between predicted probabilities and observed outcomes.

Missing data handling: The proportion of missing values for each candidate variable was reported. During the data cleaning and eligibility screening phase, any participant with missing data exceeding 5% across all candidate predictors was excluded to ensure the relative completeness of the dataset. For sporadic missing values remaining prior to modeling, imputation was performed after stratifying the dataset into training and testing sets (8∶2 ratio). Continuous variables were imputed with the median of the training set, while categorical variables were imputed with the mode of the training set. These imputation rules, derived solely from the training set, were applied to the testing set. Notably, undetected laboratory indicators were not arbitrarily coded as “normal” or “negative.”

Sensitivity analysis for class imbalance: Given the limited number of positive CNSI cases, sensitivity analyses were conducted exclusively within the training set using three resampling strategies: oversampling (targeting a 1∶1 ratio), mixed sampling (approximating a 1∶1 ratio), and mixed sampling (approximating a 2∶1 ratio). The distribution of the original hold-out testing set remained unchanged throughout the analysis to reflect prevalence.

Interpretability analysis: Model interpretability was assessed using Shapley Additive Explanations (SHAP). Both global and local (individual-level) explanations were generated to elucidate the direction and magnitude of each variable's contribution to the model predictions. These findings were subsequently cross-validated with the OR derived from the multivariable logistic regression model to ensure consistency between algorithmic outputs and clinical epidemiology.

Data and code availability: The de-identified dataset and source code supporting the findings of this study have been made publicly available on GitHub at: https://github.com/yaotianhua0924/Central-Nervous-System-Infections.

### 2.4 Theoretical approval

This study was approved by the Scientific Ethics Committee of Xiamen Children's Hospital [Approval No.: Xia'er Ke Lun Shen (20250211-2)] and was exempt from informed consent.

## 3 Results

### 3.1 Comparison of baseline characteristics

A total of 140 pediatric patients were enrolled in this study, including 28 in the CNSI group and 112 in the FS group. Among them, 92 were male (65.7%) and 48 were female (34.3%), with a male-to-female ratio of 1.9:1; the median age was 3.2 (1.5, 5.3) years.

Univariate analysis revealed significant intergroup differences in 23 variables (*P* < 0.05), including total length of hospital stay, PICU length of stay, status epilepticus, bacterial species, use of use of anxiolytic drugs, use of antiviral drugs, use of sedatives, use of intracranial pressure-lowering drugs, number of seizures, positive neurological signs, headache, vomiting, whether lumbar puncture was performed, N%, L%, RBC, LAC, CA, PO_2_, SO_2_, LAC, EEG abnormalities, and craniocerebral computed tomography (CT) abnormalities (Table 1).

**Table 1:** Comparison of baseline characteristics between the Two groups of pediatric patients.

项目	Encephalitis group	Febrile seizures group	P
Sexual(Man:Woman)	21 (75%)/7 (25%)	72 (63.4%)/41 (36.6%)	0.247
Age (years)	3.675 (1.30, 8.15)	3.200 (1.50, 4.87)	0.389
Total hospital (days)	10.00 (7.00, 14.00)	5.00 (5.00, 6.00)	<0.001
Total PICU (days)	2.50 (1.00, 3.00)	0.00 (0.00, 0.00)	<0.001
Time from fever onset to convulsion (hours)	9.50 (4.00, 24.00)	8.50 (4.00, 20.00)	0.484
Heat peak (℃)	39.00 (38.75, 39.45)	39.30 (39.000, 39.90)	0.055
Convulsive fever (℃)	38.90 (38.60, 39.10)	39.00 (38.65, 39.50)	0.1203
Duration of convulsions (minutes)	2.00 (0.75, 5.00)	2.00 (1.00, 4.00)	0.9054
Convulsions appearance(Comprehensive:Partial)	24 (85.7%)/4 (14.3%)	104 (92.8%)/8 (7.2%)	0.258
Febrile seizures(number)	1.00 (1.00, 2.00)	1.00 (1.00, 1.00)	0.049
Status epilepticus(Yes:No)	5 (17.9%)/23 (82.1%)	3 (2.7%)/109 (97.3%)	0.008
Glasgow Coma Scale(Scale)	15.00 (12.00, 15.00)	15.00 (13.00, 15.00)	0.727
Babinski(Positive:Negative)	6 (21.4%)/22 (78.6%)	0 (0.0%)/112 (100.0%)	<0.001
History of FS(Yes:No)	10 (35.7%)/18 (64.3%)	46 (41.0%)/66 (59.0%)	0.605
Family history of FS(Yes:No)	2 (7.1%)/26 (92.9%)	16 (14.3%)/96 (85.7%)	0.528
Headache(Yes:No)	5 (17.9%)/23 (82.1%)	4 (3.6%)/108 (96.4%)	0.016
Vomiting(Yes:No)	9 (32.1%)/19 (67.9%)	12 (10.7%)/100 (89.3%)	0.014
Delivery by cesarean section(Yes:No)	11 (39.3%)/17 (60.7%)	36 (32.1%)/76 (67.9%)	0.474
Lumbar puncture(Yes:No)	26 (92.9%)/2 (7.1%)	10 (8.9%)/102 (91.1%)	<0.001
Anxiolytic drugs use(Yes:No)	18 (64.3%)/10 (35.7%)	18 (16.1%)/94 (83.9%)	<0.001
Gluconate use(Yes:No)	21 (75.0%)/7 (25.0%)	8 (7.1%)/104 (92.9%)	<0.001
Antibiotics use(Yes:No)	17 (60.7%)/11 (39.9%)	58 (51.8%)/54 (48.2%)	<0.001
Antiviral drugs use(Yes:No)	25 (89.3%)/3 (10.7%)	40 (35.7%)/72 (64.3%)	<0.001
Bacteria(Unknown:Coccidioides immitis:Salmonella typhimurium:Streptococcus pneumoniae:Haemophilus influenzae:Mycoplasma pneumoniae:Group A Streptococcus:Klebsiella pneumoniae)	20 (71.4%)/0 (0.0%)/0 (0.0%)/1 (3.6%)/1 (3.6%)/3 (10.7%)/0 (0.0%)/3 (10.7%)	91 (81.3%)/2 (1.8%)/2 (1.8%)/1 (0.9%)/2 (1.8%)/11 (9.8%)/3 (2.7%)/0 (0.0%)	0.030
Virus(Unknown:Influenza A virus:Influenza B virus:Novel Coronavirus:Rhinovirus:Parainfluenza virus:Adenovirus:Coronavirus:Respiratory Syncytial Virus:Paramyxovirus:Herpes Simplex Virus)	6 (21.4%)/11 (39.3%)/3 (10.7%)/1 (3.55%)/3 (10.7%)/0 (0.0%)/1 (3.6%)/0 (0.0%)/1 (3.6%)/1 (3.6%)/1 (3.6%)	40 (35.7%)/32 (28.6%)/7 (6.2%)/3 (2.6%)/14 (12.5%)/4 (3.5%)/4 (3.5%)/3 (2.6%)/3 (2.6%)/0 (0.00%)/2 (1.8%)	0.521
Breathing(Seconds/Minutes)	29.00 (25.00, 33.00)	28.00 (25.00, 30.00)	0.632
Heart rate(Seconds/Minutes)	131.00 (116.50, 142.50)	130.00 (119.50, 145.00)	0.658
Systolic blood pressure(mmHg)	100.50 (90.50, 108.00)	96.00 (90.00, 102.00)	0.118
Diastolic blood pressure(mmHg)	56.50 (46.50, 64.50)	56.000 (52.50, 62.00)	0.333
WBC(109/L)	9.550 (6.68, 10.48)	9.120 (6.75, 12.99)	0.656
N(%)	61.564 ± 19.180	69.641 ± 13.362	0.043
L(%)	23.25 (12.75, 39.65)	17.00 (10.90, 25.90)	0.038
RBC(1012/L)	4.39 (4.22, 4.54)	4.49 (4.30, 4.73)	0.042
PLT(109/L)	261.50 (205.50, 320.00)	255.00 (212.00, 338.50)	0.773
HGB(g/L)	117.214 ± 12.773	121.152 ± 8.915	0.133
CRP(mg/L)	3.41 (1.27, 12.01)	4.60 (1.51, 11.60)	0.707
PCT(ng/mL)	0.23 (0.11, 0.73)	0.24 (0.14, 0.54)	0.855
ALT(U/L)	16.60 (13.00, 21.00)	15.60 (12.80, 18.70)	0.570
AST(U/L)	34.714 ± 12.789	35.241 ± 9.252	0.839
ALB(g/L)	41.196 ± 3.095	43.082 ± 2.476	<0.001
TBA(umol/L)	6.40 (4.05, 7.60)	6.20 (4.70, 8.25)	0.837
CR(umol/L)	32.221 ± 8.616	30.703 ± 7.278	0.343
BUN(mmol/L)	3.78 (3.36, 4.52)	3.59 (3.09, 4.47)	0.617
CK(U/L)	123.85 (93.50, 154.95)	120.05 (89.35, 155.40)	0.973
CK-MB(U/L)	27.65 (21.20, 32.85)	28.40 (22.60, 34.50)	0.408
LDH(U/L)	278.65 (242.45, 314.05)	303.35 (261.55, 356.85)	0.092
NA(mmol/L)	135.239 ± 2.798	136.181 ± 2.462	0.081
K(mmol/L)	3.922 ± 0.434	3.943 ± 0.436	0.819
CA(mmol/L)	2.14 (1.16, 2.29)	1.18 (1.13, 1.26)	<0.001
GLU(mmol/L)	5.89 (5.14, 6.95)	5.90 (5.15, 6.70)	0.628
PH	7.38 (7.35, 7.41)	7.39 (7.36, 7.42)	0.363
PO2(mmHg)	81.55 (67.60, 105.50)	60.35 (42.00, 83.85)	0.004
PCO2(mmHg)	36.643 ± 7.372	35.553 ± 6.039	0.416
SO2(%)	96.90 (93.80, 99.00)	90.85 (75.30, 97.15)	0.001
HCO3(mmol/L)	21.436 ± 2.532	21.228 ± 2.253	0.671
LAC(mmol/L)	1.10 (0.70, 1.40)	1.60 (1.05, 2.10)	0.019
Electroencephalogram(No check:abnormal:normal)	1 (3.6%)/15 (53.6%)/12 (42.9%)	5 (4.5%)22 (19.6%)/85 (75.9%)	0.001
Cranial CT(No check:abnormal:normal)	6 (21.4%)/8 (28.6%)/14 (50.0%)	54 (48.2%)/12 (10.7%)/46 (41.1%)	0.010
Cranial MRI(No check:abnormal:normal)	3(10.7%)/15(53.6%)/10(35.7%)	21(18.8%)/40(35.7%)51(45.5%)	0.206

### 3.2 Feature selection

Following univariate analysis (*α* = 0.05) to identify significant variables, post-intervention indicators variables such as lumbar puncture, use of antiviral agents, intracranial pressure-lowering agents, sedatives, and antibiotics, as well as length of hospital stay, were excluded based on clinical *a priori* criteria.Spearman correlation coefficients were calculated for the remaining nine quantitative variables; when |r| ≥ 0.70, the variable with the smaller *P*-value in univariate analysis was retained within each highly correlated pair to mitigate collinearity. Subsequently, RF and XGBoost were employed to rank variable importance. Features with a normalized importance value exceeding 0.05 were selected, resulting in four distinct feature sets: RF-Thresh (D1), XGB-Thresh (D2), Union (D3), and Intersection (D4).

**Figure 1:**
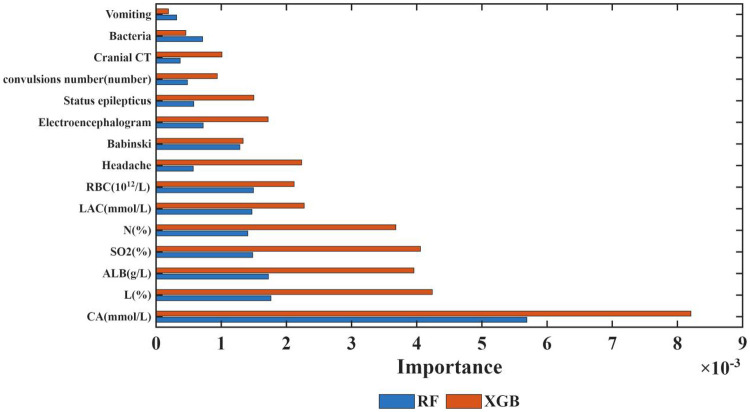
Importance ranking. RF, random fores; XGB, extreme gradient boosting.

Correlation analysis revealed a strong collinearity between PO_2_ and Ca (|r| = 0.955). Given that Ca demonstrated a more significant univariate association with the outcome, PO_2_ was excluded prior to feature selection. Combining the remaining quantitative variables with 7 categorical variables showing significant intergroup differences, a total of 15 variables were included for importance ranking. The ranking results indicated that RF selected 8 variables, while XGBoost selected 9 variables, yielding 10 variables in the union set and 7 in the intersection set. Comparative analysis of these four datasets was performed using LogitBoost, configured with the following hyperparameters: NumLearningCycles = 320; LearnRate = 0.09; MinLeafSize = 12; MaxNumSplits = 6; and KFold = 5.The results demonstrated that the Intersection dataset (D4) achieved the optimal performance, with an AUC of 0.795 (95% CI: 0.547, 0–960). Consequently, the final prediction model incorporated the following 10 variables: Ca, L(%), ALB, RBC, SO_2_, LAC, N(%), Babinski sign, Headache, and EEG (Figure 1).

### 3.3 Interpretability model

**Figure 2:**
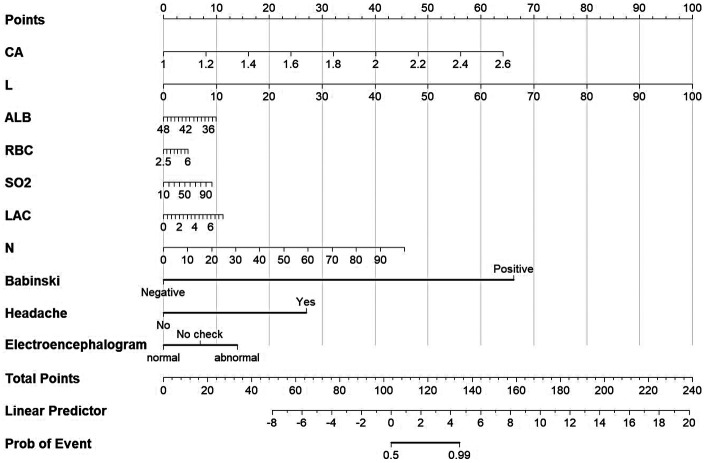
Nomograms (based on logistic regression).

Based on the 10 predictors selected via the Union strategy, a multivariable logistic regression model was constructed to predict CNSI, and a nomogram was developed for individualized risk assessment. Multivariate analysis identified serum Ca as an independent risk factor for CNSI (OR = 380.733, 95% CI: 21.773–6, 657.712, *P* < 0.001), indicating that lower Ca levels are significantly associated with an increased risk of CNSI. Headache was also significantly associated with CNSI (OR = 0.018, 95% CI: 0.002–0.200, *P* = 0.001), likely reflecting elevated intracranial pressure. Additionally, EEG abnormalities showed a trend toward association with CNSI risk (*P* = 0.068). The remaining variables—including L(%), ALB, RBC, SO_2_, LAC, N(%), and Babinski sign—did not demonstrate independent statistical significance in the multivariable model (*P* > 0.05) (Figure 2).

**Figure 3:**
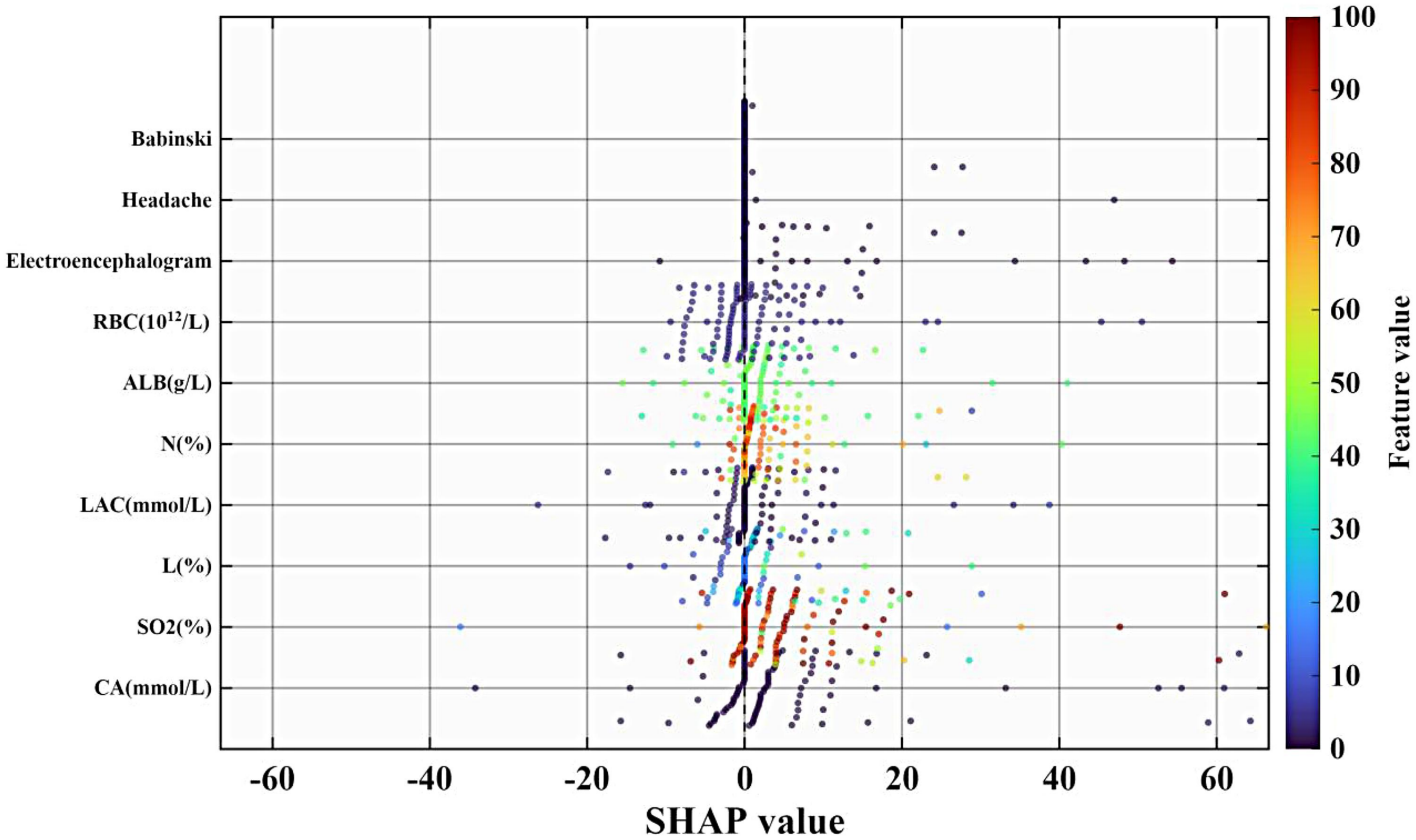
SHAP swarm plot.

A LogitBoost gradient boosting model was further developed, and SHAP were employed to quantitatively interpret the model outputs. Ranked by mean absolute SHAP values, the top contributing variables were identified as: Ca (15.121), SO_2_(8.169), RBC (4.754), L(%) (4.740), and LAC (4.067), followed by ALB (3.960), N(%) (3.826), and EEG (3.332). Collectively, Ca, SO_2_, and L(%) accounted for approximately 56.8% of the model's predictive variance, with Ca emerging as the dominant driver (mean SHAP = 12.480, SD = 28.590) (Figure 3).

Analysis of SHAP value directions and distributions revealed the following insights: Ca exhibited a positive SHAP contribution in 62.50% of samples, indicating that lower Ca levels are associated with an increased risk of CNSI. Both SO_2_(72.32% positive) and N(%) (69.64% positive) demonstrated a graded relationship with CNSI risk across different value ranges, characterized by distinct separation in SHAP distributions between high and low levels. ALB also displayed a biphasic pattern: higher values tended to confer protection, whereas lower values suggested elevated risk, with 56.25% of samples showing a positive SHAP contribution. LAC maintained a relatively low mean absolute SHAP value, primarily exerting a negative corrective effect, which appears to provide risk calibration for extreme predictions.Conversely, the Babinski sign and Headache exhibited minimal marginal contributions in the nonlinear model, with mean absolute SHAP values of 0.017 and 1.358, respectively (Figure 3).

### 3.4 Comparison of model performance

Within the framework of an 8∶2 stratified train-test split, seven predictive models were constructed and compared: LR, LASSO, Ridge, SVM, BPNN, RF, and LogitBoost—to compare the predictive performance of different algorithms under the conditions of small sample size and class imbalance. Given the limited number of positive CNSI cases (*n* = 28), this analysis was positioned as an exploratory model comparison. On one hand, machine learning models such as RF and LogitBoost were retained, with model complexity strictly controlled by limiting tree depth, leaf node samples, learning rates, and iteration counts. On the other hand, regularized logistic models (LASSO and Ridge) were included as baseline comparators suitable for small-sample scenarios.

**Figure 4:**
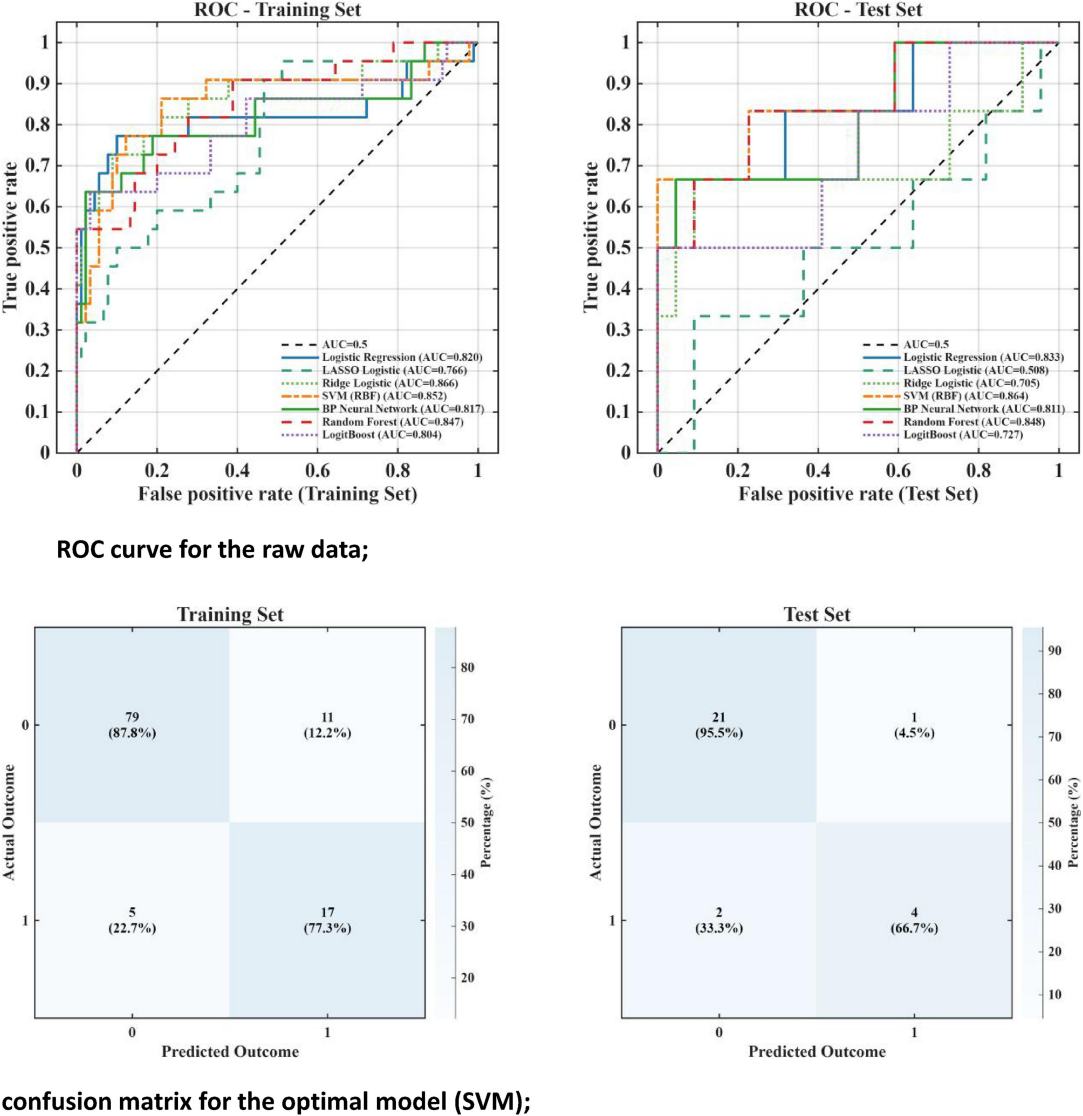
ROC curve for the raw data and confusion matrix for the optimal model (SVM). **(A)** ROC curve for the raw data; **(B)** confusion matrix for the optimal model (SVM).

Model training was conducted exclusively within the training set. Training set performance was evaluated using OOF predictions derived from 5-fold cross-validation to prevent information leakage and mitigate overfitting. Test set metrics were generated from the final models fitted on the entire training cohort.To quantify the uncertainty inherent in small-sample evaluations, AUC and its 95% CI were estimated via repeated cross-validation (aggregating OOF predictions). Other metrics—including accuracy, precision, sensitivity, specificity, and the F1-score—were calculated using the optimal threshold determined by maximizing the Youden index on the training set OOF predictions.Test set results demonstrated that SVM achieved the highest discrimination (AUC = 0.864, 95% CI: 0.625–1.000), followed by RF (AUC = 0.848, 95% CI: 0.596–1.000) and LR (AUC = 0.833, 95% CI: 0.578–1.000). BPNN (AUC = 0.811, 95% CI: 0.500–1.000) and LogitBoost (AUC = 0.727, 95% CI: 0.404–1.000) showed intermediate performance, whereas Ridge (AUC = 0.705, 95% CI: 0.287–1.000) and LASSO (AUC = 0.508, 95% CI: 0.174–0.833) performed relatively poorly. It is imperative to emphasize that the wide 95% CIs across all models are a natural consequence of Bootstrap resampling within the small test set (*n* = 28). Therefore, these point estimates should not be directly extrapolated to define clinically applicable thresholds. Considering both AUC and accuracy, the SVM model exhibited the most stable comprehensive predictive performance on the test set (Table 2, Figure 4).

**Table 2:** Model comparison results for the original dataset.

Model	Split	Accuracy	Precision	Sensitivity	Specificity	F1	AUC_95 CI
LR	Train	0.875	0.654	0.773	0.900	0.708	0.820 (0.726–0.914)
	Test	0.893	0.800	0.667	0.955	0.727	0.833 (0.739–0.927)
LASSO	Train	0.598	0.317	0.909	0.522	0.471	0.766 (0.646–0.886)
	Test	0.607	0.273	0.500	0.636	0.353	0.508 (0.388–0.628)
Ridge	Train	0.875	0.667	0.727	0.911	0.696	0.866 (0.760–0.972)
	Test	0.821	0.600	0.500	0.909	0.545	0.705 (0.599–0.811)
SVM	Train	0.857	0.607	0.773	0.878	0.680	0.852 (0.757–0.947)
	Test	0.893	0.800	0.667	0.955	0.727	0.864 (0.769–0.959)
BPNN	Train	0.911	0.875	0.636	0.978	0.737	0.817 (0.719–0.915)
	Test	0.857	0.999	0.333	0.999	0.500	0.811 (0.713–0.909)
RF	Train	0.821	0.536	0.682	0.856	0.600	0.847 (0.758–0.936)
	Test	0.821	0.571	0.667	0.864	0.615	0.848 (0.759–0.937)
LogitBoost	Train	0.902	0.824	0.636	0.967	0.718	0.804 (0.682–0.926)
	Test	0.786	0.500	0.500	0.864	0.500	0.727 (0.605–0.849)

LR, logistic regression; LASSO, least absolute shrinkage and selection operator; Ridge, ridge regression; SVM, support vector machines; BPNN, backpropagation neural networks; RF, random forest.

### 3.5 Sensitivity analysis for class balancing

Given that this study included only 28 CNSI-positive samples and exhibited significant class imbalance, we conducted a class-balanced sensitivity analysis on the four models (LR, SVM, BPNN, and RF) that achieved an AUC ≥ 0.8 on the original test set, based on the 10 predictive variables identified through joint screening.

**Figure 5:**
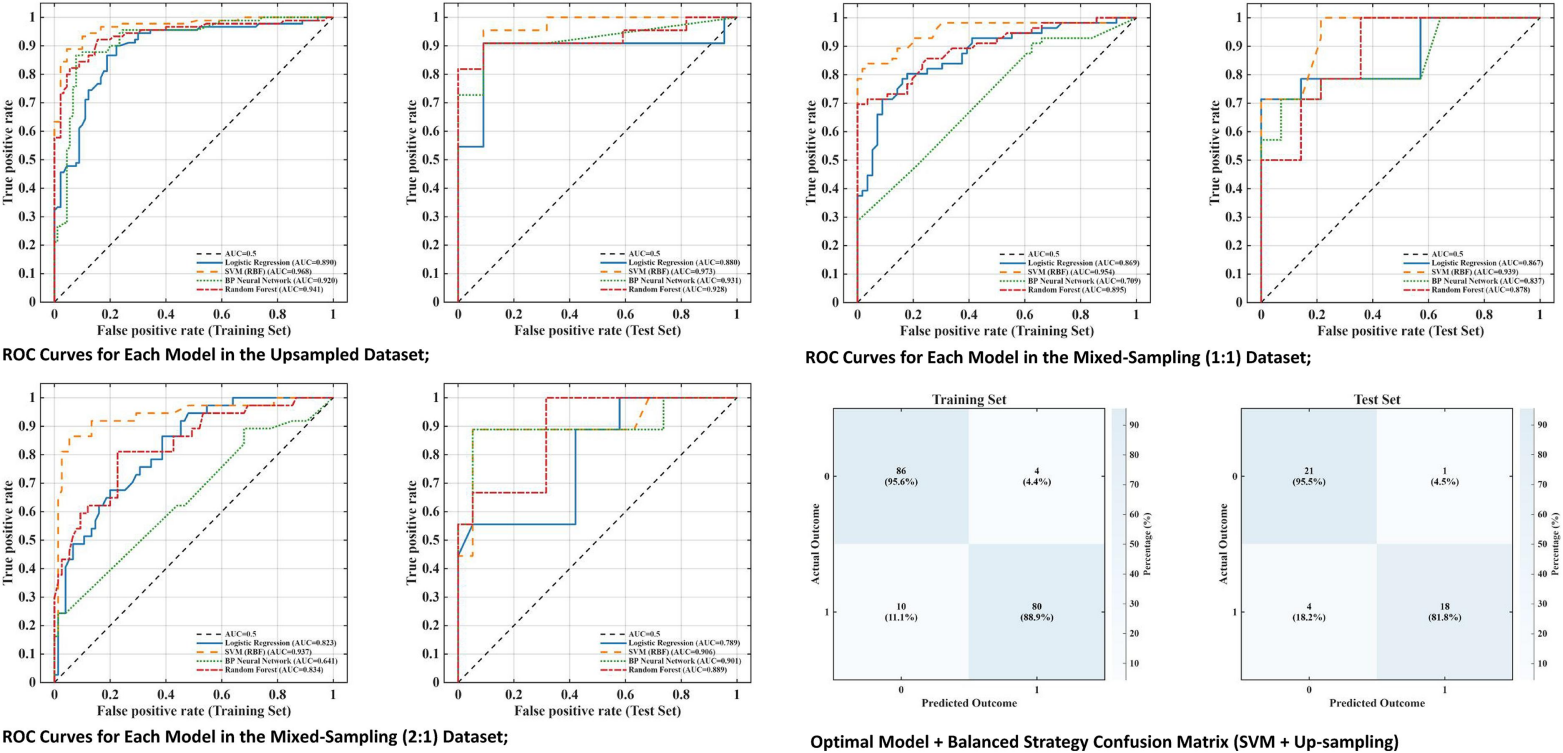
ROC curves for various models on the upsampled, mixed-sampling (1:1), and mixed-sampling (2:1) datasets, and the confusion matrix for the optimal model combined with the balancing strategy (SVM + upsampling). **(A)** ROC Curves for Each Model in the Upsampled Dataset; **(B)** ROC Curves for Each Model in the Mixed-Sampling (1:1) Dataset; **(C)** ROC Curves for Each Model in the Mixed-Sampling (2:1) Dataset; **(D)** Optimal Model + Balanced Strategy Confusion Matrix (SVM + Up-sampling).

Three specific resampling strategies were implemented: (1) up-sampling to a 1:1 ratio (112:112); (2) mixed sampling—maintaining the total sample size while approximating a 1:1 ratio (70:70); (3) mixed sampling—target ratio of 2:1 (46:94). For each strategy, an 8:2 stratified division, 5-fold OOF evaluation of the training set, and 1,000 bootstrap resamples were used to calculate the AUC and its 95% CI (Table 3, Figure 5). The test set results showed that the SVM model performed best under upsampling conditions, with an AUC of 0.973 (95% CI: 0.935–0.990). These results indicate that after excluding the three model categories with insufficient discriminative power in the original test set, RF, SVM, and BPNN still maintained moderately high and relatively consistent predictive performance (AUC approximately 0.85–0.95) under different balancing strategies, suggesting that the discriminative information retained through prior feature selection possesses good transferability; simultaneously, LR performed similarly to the complex models, supporting the study's design of a parallel comparison of multiple algorithms within an exploratory framework (Table 3, Figure 5).

**Table 3:** Model evaluation results for the validation dataset.

SamplingMethod	Model	Split	Acc	Pre	Sen	Spe	F1	AUC_95 CI
Upsample_Equal_1 to 1	LR	Train	0.839	0.802	0.900	0.778	0.848	0.890 (0.845, 0.935)
		Test	0.886	0.870	0.909	0.864	0.889	0.880 (0.835, 0.925)
	SVM	Train	0.922	0.952	0.889	0.956	0.920	0.968 (0.951, 0.985)
		Test	0.886	0.947	0.818	0.955	0.878	0.973 (0.956, 0.990)
	BPNN	Train	0.883	0.897	0.867	0.900	0.881	0.920 (0.883, 0.957)
		Test	0.886	0.870	0.909	0.864	0.889	0.931 (0.894, 0.968)
	RF	Train	0.872	0.947	0.789	0.956	0.861	0.941 (0.910, 0.972)
		Test	0.909	0.999	0.818	0.999	0.900	0.928 (0.897, 0.959)
Mixed_KeepTotal_Approx1 to 1	LR	Train	0.813	0.818	0.804	0.821	0.811	0.869 (0.813, 0.925)
		Test	0.821	0.909	0.714	0.929	0.800	0.867 (0.811, 0.923)
	SVM	Train	0.893	0.958	0.821	0.964	0.885	0.954 (0.930, 0.978)
		Test	0.821	0.909	0.714	0.929	0.800	0.939 (0.915, 0.963)
	BPNN	Train	0.634	0.623	0.679	0.589	0.650	0.718 (0.675, 0.761)
		Test	0.821	0.909	0.714	0.929	0.800	0.837 (0.794, 0.880)
	RF	Train	0.813	0.889	0.714	0.911	0.792	0.895 (0.862, 0.928)
		Test	0.750	0.818	0.643	0.857	0.720	0.878 (0.845, 0.911)
Mixed_TargetRatio_2 to 1	LR	Train	0.696	0.525	0.865	0.613	0.653	0.823 (0.759, 0.887)
		Test	0.679	0.500	0.889	0.579	0.640	0.789 (0.725, 0.853)
	SVM	Train	0.911	0.865	0.865	0.933	0.865	0.937 (0.896, 0.978)
		Test	0.893	0.800	0.889	0.895	0.842	0.906 (0.865, 0.947)
	BPNN	Train	0.732	0.818	0.243	0.973	0.375	0.639 (0.585, 0.693)
		Test	0.893	0.800	0.889	0.895	0.842	0.901 (0.847, 0.955)
	RF	Train	0.741	0.577	0.811	0.707	0.674	0.834 (0.776, 0.892)
		Test	0.821	0.750	0.667	0.895	0.706	0.889(0.831, 0.947)

LR, logistic regression; SVM, support vector machines; BPNN, backpropagation neural networks; RF, random forest.

## 4 Discussion

CNSI is one of the leading causes of neurological injury in children. It has a high incidence and mortality rate and is the most common cause of hospitalization among children in many developing countries. Without early and timely treatment, it often leads to serious complications such as brain abscesses, hydrocephalus, brain developmental abnormalities, and epilepsy, significantly impacting the health and well-being of affected children (11, 12). Clinically, CNSI presents a formidable diagnostic challenge due to its nonspecific manifestations, with fever accompanied by seizures being the most frequent initial presentation. This clinical overlap makes it particularly difficult to distinguish CNSI from typical FS (13). Studies have found that one in four children with bacterial meningitis experiences febrile seizures, and complex febrile seizures are also considered a risk factor for bacterial meningitis (14, 15). Therefore, early recognition of CNSI and prompt treatment are key to preventing adverse neurological sequelae in affected children.

Machine learning has been increasingly utilized to assist in assessing the risk of pediatric infections and meningitis, with extensive research covering areas such as serious bacterial infections, sepsis, meningitis, risk stratification in febrile children, and predicting emergency department outcomes. Traditional predictive models for childhood CNSI—such as the Children's Bacterial Meningitis Score, the Rochester Criteria, and prognostic nomograms for bacterial meningitis—have demonstrated significant clinical value in diagnosing bacterial meningitis (8–10). However, these models largely rely on cerebrospinal fluid (CSF) analysis for confirmation, which is inherently invasive and limits their generalizability in primary healthcare settings, thereby hindering the early screening of high-risk CNSI cases.

This study aims to use machine learning methods to integrate early-stage data—including clinical characteristics and routine laboratory test results—to develop an exploratory model for distinguishing between pediatric CNSI and FS based on variables available at the time of initial diagnosis. The goal is to provide a preliminary reference for early risk stratification of pediatric CNSI and improve patient outcomes.

This study compared seven predictive models, including LR, LASSO, Ridge, SVM, BPNN, RF, and LogitBoost. The results demonstrated that the SVM model achieved the optimal performance, with an AUC of 0.864 (95% CI: 0.625–1.000). This finding aligns with the established advantages of machine learning algorithms in processing complex medical data. Compared to traditional statistical models, the SVM algorithm is particularly adept at handling small clinical sample sizes, nonlinear relationships, and high-dimensional data, offering high accuracy, robust generalization capability, and stable performance (16–18). The high AUC value observed with the SVM model in this study indicates that integrating early-stage indicators—such as clinical features and routine laboratory findings—via machine learning can significantly enhance the discriminatory ability between CNSI and FS, thereby providing a powerful auxiliary tool for clinical decision-making.

Following univariate screening, Spearman correlation analysis, multicollinearity assessment, and feature selection via RF and XGBoost importance ranking (threshold > 0.05), a union feature set was established, ultimately identifying ten core predictors: Ca, L(%), ALB, RBC, SO_2_, LAC, N(%), Babinski sign, Headache, and EEG.SHAP analysis (19, 20) identified Ca, SO_2_, and L(%) as the top contributors to CNSI prediction. Serum Ca emerged as the strongest driver (mean SHAP = 12.480, SD = 28.590). Analysis of SHAP value distributions revealed a positive contribution in 62.50% of samples, indicating that lower Ca levels are associated with an increased risk of CNSI. This aligns with the pathophysiology of CNSI, wherein neuronal injury disrupts Ca homeostasis. Hypocalcemia lowers the neuronal excitation threshold, potentially triggering seizures. Moreover, low serum Ca is often a marker of poor prognosis in severe CNSI, likely linked to mitochondrial dysfunction and pump failure; intracellular Ca overload can activate proteases and phospholipases, leading to neuronal death and synaptic dysfunction (21–23).

SO_2_ also demonstrated a significant positive contribution in 72.32% of samples, exhibiting a gradient relationship with CNSI risk across its value range. Both excessively high and low SO_2_ levels were indicative of elevated risk. Unlike simple FS, CNSI patients often suffer from respiratory center involvement or ventilation dysfunction due to severe seizures, leading to decreased PO_2_ and SO_2_ alongside elevated LAC (24–26). Conversely, abnormally high SO_2_ may indicate the administration of high-concentration oxygen support, suggesting severe illness and suppressed spontaneous respiration. Hyperoxia-induced blood-brain barrier (BBB) damage may further increase CNSI risk (27, 28). In this study, CNSI patients exhibited significantly lower PO_2_ and SO_2_ than FS patients, suggesting that unexplained hypoxemia at admission should prompt clinicians to prioritize the evaluation of CNSI.

Regarding leukocyte differentials, the CNSI group presented with significantly lower N(%) and higher L(%) compared to the FS group. This pattern likely reflects the high prevalence of viral etiologies (such as influenza virus and novel coronavirus eta.) in our cohort, as viral infections typically induce reactive lymphocytosis, consistent with established hematological profiles of viral encephalitis (29, 30).

The model identified ALB as a significant risk factor for CNSI, an indicator often underestimated in pediatric critical illness. Severe infections can suppress hepatic ALB synthesis and increase vascular permeability, leading to ALB leakage. ALB not only reflects disease severity but may also exacerbate the condition by affecting drug metabolism and immune function (31–34).Studies have found that low ALB impairs the blood-brain barrier, increases its permeability, and facilitates the entry of pathogens and inflammatory mediators into the central nervous system, thereby exacerbating encephalitis; monitoring ALB levels aids in identifying children with CNSI (35, 36).

SHAP analysis revealed that the Babinski sign and Headache contributed minimally to the nonlinear model predictions, with mean absolute SHAP values of 0.017 and 1.358, respectively. While a positive Babkin sign indicates involvement of the corticospinal tract and is a hallmark of severe neurological injury, previous studies have reported its low sensitivity for predicting CNSI (37, 38). Consequently, a negative Babkin sign cannot reliably exclude CNSI, limiting its utility for early risk stratification in the acute setting. While headache often indicates elevated intracranial pressure and can aid in the clinical diagnosis of CNSI, its utility for early risk stratification in children is limited. Headache is a non-specific symptom influenced by various confounding factors—such as fever, sinusitis, or emotional distress—and younger children are often unable to articulate this symptom accurately (39, 40). Consequently, despite its pathophysiological relevance, headache contributed minimally to the model's predictive power (mean absolute SHAP = 1.358).

EEG is a noninvasive technique that records cerebral bioelectrical activity via scalp electrodes, traditionally utilized for epilepsy diagnosis but increasingly adopted for the noninvasive evaluation of CNSI. During CNSI, neuroinflammation, cerebral edema, and microvascular spasm disrupt neuronal metabolism and synchronization, leading to characteristic EEG abnormalities (41, 42). However, the utility of EEG for early risk stratification in children is limited. Its application requires specialized equipment and trained personnel, and critically, EEG often yields normal results in the early stages of CNSI when parenchymal injury is still mild, thereby reducing its sensitivity for early detection.

## 5 Conclusion

This single-center retrospective study developed an exploratory predictive model to differentiate CNSI from FS in children presenting with fever and convulsions. Leveraging machine learning, we integrated readily accessible clinical and laboratory data obtained during the initial visit or early admission. Among the combined feature sets and various algorithms evaluated, the SVM demonstrated relatively superior discriminatory performance in internal validation. Furthermore, SHAP analysis identified CA, SO_2_, and L% as significant contributors to the model's predictive power. At present, this model should be regarded merely as a preliminary reference for the early risk stratification of CNSI. Its clinical applicability warrants further validation and calibration through future multicenter, prospective cohort studies.

## 6 Limitations and prospects

This study has several limitations that warrant consideration. First, the inherent nature of the retrospective design introduces potential selection bias and incomplete data capture. Notably, laboratory tests were not routinely performed for all children with FS, resulting in non-random missingness for certain variables. Second, as a single-center study conducted within the Chinese population, the generalizability of our model to other ethnicities or healthcare settings remains uncertain. Third, despite our efforts, the exact timing of blood collection relative to clinical events was difficult to fully ascertain, potentially leaving residual temporal bias. Fourth, internal evaluation metrics cannot substitute for external validation; thus, we cannot yet claim superiority over established clinical decision rules, such as the Rochester criteria. Finally, the limited number of positive events (CNSI cases) may affect the model's stability.

Moving forward, we aim to establish multicenter collaborations to expand our sample size. We also plan to develop an intelligent early warning system based on real-time extraction of electronic health record (EHR) data. By embedding this model into the clinical workflow, we seek to enable real-time automated triage for children with FS and provide dynamic risk warnings for CNSI.

## Data Availability

The raw data supporting the conclusions of this article will be made available by the authors, without undue reservation.
